# EGFR overexpression is not common in patients with head and neck cancer. Cell lines are not representative for the clinical situation in this indication

**DOI:** 10.18632/oncotarget.25656

**Published:** 2018-06-22

**Authors:** Sami Sebastian Khaznadar, Martin Khan, Elke Schmid, Sebastian Gebhart, Eva-Tessina Becker, Thomas Krahn, Oliver von Ahsen

**Affiliations:** ^1^ Biomarker Research, Bayer AG, 13353 Berlin, Germany; ^2^ Charite, Berlin, 13353 Berlin, Germany; ^3^ Present address: Klinikum Dahme-Spreewald GmbH, 15711 Königs-Wusterhausen, Germany; ^4^ Present address: University Bonn, 53113 Bonn, Germany

**Keywords:** HNSCC, Erlotinib, IHC, MSD, tyrosine kinase inhibitor

## Abstract

**Background:**

Based on expression data, Epidermal Growth Factor Receptor (EGFR) emerged as therapeutic target in Head and Neck Cancer but clinical efficacy of EGFR inhibitors was very limited. We reinvestigated the EGFR expression and activation status necessary for response in cell lines and compared that to clinical samples.

**Methods:**

Clinical samples of head and neck squamous cell carcinoma (HNSCC, n=63), mostly from late stage (IV) and poorly or undifferentiated character and cultured cell lines (n=14) were tested by immunohistochemistry (IHC) (n=55) and sandwich immunoassays (n=63) for expression and phosphorylation of EGFR (Tyrosine-1173). Response of 14 different HNSCC cell lines to Erlotinib was tested in proliferation assays.

**Results:**

Most HNSCC cell lines respond to Erlotinib. EGFR is phosphorylated in these cell lines. Resistant cell lines display very low level EGFR expression and phosphorylation. EGFR activity in clinical samples is significantly below that observed in cell lines. In clinical samples, EGFR is not overexpressed on the single cellular level. We show similar levels of EGFR expression in growing keratinocytes and tumor cells.

**Conclusions:**

Cell lines are not representative of the clinical situation in HNSCC. Larger studies should investigate whether patient subgroups with activating EGFR mutations or overexpression can be identified.

## INTRODUCTION

Squamous cell carcinomas of the upper aerodigestive tract, usually summarized as head and neck squamous cell carcinoma (HNSCC), represent the sixth most common cancer in the world [1]. Survival rates have not been improved during the last decades, many patients develop recurrence and metastatic disease leading to 5 year survival rates below 50%. Conventional treatment strategies including surgery, radiation and chemotherapy are effective in only 50% of the patients and are associated with significant toxicities, highlighting the need for additional treatments [2]. Based on its broad expression in up to 90% of the HNSCC cases, epidermal growth factor receptor emerged as potential target for targeted therapies [3]. The relevance of EGFR was supported by its prognostic value predicting time to relapse and overall survival [4]. The first approved targeted treatment for HNSCC is the anti-EGFR antibody Cetuximab which showed clinical efficacy in combination with cisplatin [5] and radiotherapy [6]. However, no predictive value for Cetuximab use could be derived from data on EGFR copy number, protein expression or mutation [7, 8]. This is in line with the broad expression of EGFR and antibody dependent cellular cytotoxicity (ADCC) as mode of action.

In addition to Cetuximab, the EGFR TKIs Erlotinib and Gefitinib were tested for their efficacy in clinical trials. However, the clinical experiences with EGFR TKIs in HNSCC were disappointing: A single arm study on Gefitinib monotherapy in recurrent or metastatic HNSCC observed a response rate of 10.6% [9], a multicenter phase 2 study of Erlotinib monotherapy obtained an objective response rate of 4.3% and a disease stabilization for 4 months in 38% of the patients but lacked a clear conclusion due to the lack of a control arm [10]. Addition of Erlotinib to radio-chemotherapy did not increase the CRR or PFS [11]. Addition of Gefitinib in a single arm phase 2 study did not improve the outcome of radio-chemotherapy compared to a historical control group [12].

Several groups have described factors which may cause resistance to EGFR inhibition and found Cyclin D1 overexpression [13], ErbB2, ErbB3 signaling [14] and EMT [15, 16] associated with resistance but the question how precisely the cell culture models match the clinical situation was not addressed.

Therefore, we re-evaluated EGFR as target in HNSCC in different cell lines and compared the properties of HNSCC cell lines with fresh tumor biopsies in order to validate the use of cell lines as representative of the clinical situation.

Usually, expression of therapeutic targets in tumor samples is done by immunohistochemistry. We extended the usual investigation of target expression with testing EGFR expression and also its phosphorylation by MSD immunoassays. Due to the use of two independent antibodies, the specificity of these assays is very high and also the dynamic range much wider compared to immunohistochemistry.

Tyrosine-1173 is one of the tyrosine residues in the cytoplasmic tail of EGFR which are phosphorylated in response to receptor activation. Upon activation of EGFR, several tyrosines become phosphorylated with same kinetics eg. nicely shown by Hsu and colleagues [17]. To our knowledge there is no differential regulation of different phosphorylation sites in the cytoplasmic tails of RTKs. Together with phosphotyrosine-992 and -1148, phosphotyrosine-1173 provides the docking site for the adaptor proteins SHC and Grb2 which mediate the recruitment and activation of ras downstream of EGFR activation [18, 19].

Therefore, the analysis of EGFR phosphorylation is especially useful to test for the functional relevance of the protein.

## RESULTS

### Erlotinib sensitivity of HNSCC cell lines

We first tested the response to Erlotinib in cell culture. We used 14 HNSCC cell lines and two control cell lines, KPL-4 as negative control without EGFR expression and HCC-827, a NSCLC cell line with an activating mutation in the EGFR gene. Table 1 shows that most of the cell lines responded well to Erlotinib with IC50 values in the submicromolar range. In order to minimize the risk of non-specific effects Erlotinib was used in a maximum concentration of 3 μM. For the investigation of specific effects this concentration should be sufficient since the K_i_ of Erlotinib at EGFR is 17.5 nM [20]. Only two of the HNSCC cell lines did not show a clear response at 3 μM Erlotinib. According to the expectation, KPL4 cells did not respond to Erlotinib while HCC-827 showed 95% efficacy with high potency. Overall, 12 of 14 HNSCC lines responded to Erlotinib treatment supporting the use of EGFR inhibitors in HNSCC. We also tested the possible impact of EGF concentration in the cell culture medium. Addition of 1 ng/ml EGF to the medium did not change the response. The EGF concentration in regular medium is therefore not limiting the cell growth.

**Table 1:** HNSCC cell lines respond to Erlotinib

Cell line	IC50 (nM)	Efficacy (%)
Esophagus Tumor KYSE-30	854	80
Esophagus Tumor KYSE-70	>3000	30
Esophagus Tumor KYSE-150	190	76
Esophagus Tumor KYSE-140	384	80
Esophagus Tumor KYSE-510	>3000	30
Tongue Tumor SCC-4	265	47
Tongue Tumor SCC-9	352	95
Tongue Tumor SCC-15	450	100
Tongue Tumor SCC-25	234	99
Tongue Tumor CAL 27	309	88
Tongue Tumor CAL 33	712	85
Tongue Tumor HSC-3	1710	68
Pharynx Tumor Metastasis Detroit 562	431	67
Pharynx Tumor FaDu	568	68
Lung Tumor HCC827	18	95
Breast Tumor Metastasis KPL-4	>3000	32

In a three day proliferation assay, the majority of the tested cell lines responded to Erlotinib but with variable sensitivity and efficacy. The EGFR-negative control cell line KPL-4 and two HNSCC lines responded weakly to Erlotinib. The EGFR mutated NSCLC cell line HCC827 showed highest sensitivity to EGFR inhibition.

### Expression and phosphorylation status of EGFR in cell lines

**Figure 1:**
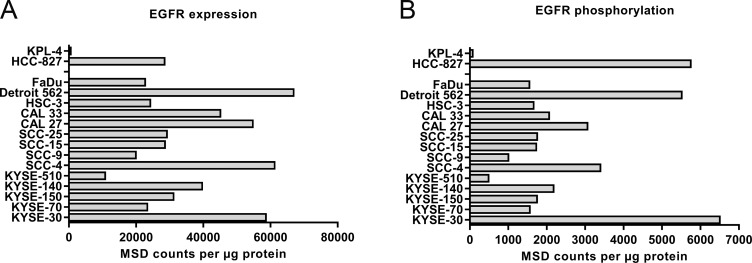
Expression **(A)** and phosphorylation **(B)** of EGFR in 14 different HNSCC cell lines and two controls (KPL-4 and HCC-827). EGFR expression in lysates of exponentially growing cells containing 20 μg protein was tested by sandwich-immunoassay, mesoscale discovery (MSD). The signaling activity of EGFR was tested by determination of the phosphotyrosine-1173 in the activation-loop of the kinase also by MSD assay. Expression and phosphorylation levels correlate with Pearson r=0.74 (see also Supplementary Figure 1).

We then characterized the cell lines for expression and activity levels of EGFR (Figure 1).

Upon activation of EGFR, several tyrosine residues in the cytoplasmic tail become phosphorylated with same kinetics [17]. These phosphotyrosines provide the docking site for the adaptor proteins which mediate the activation of the ras-raf-MEK-ERK pathway. This is the major signaling activity of EGFR and therefore a good readout for EGFR activity. For this reason, we used the MSD duplex assay for total and phosphotyrosine-1173 EGFR.

All cell lines tested expressed EGFR with the exception of the negative control KPL-4. Expression and phosphorylation correlated well for the HNSCC cell lines, only the NSCLC control HCC-827 formed an outlier with higher phosphorylation due to the activating mutation (see also Supplementary Figure 1). The non-responder cell line KYSE-510 had the lowest EGFR expression among the HNSCC cells tested. The analysis of tyrosine-1173 phosphorylation gave a similar picture. However, the second non-responder cell line, KYSE-70 did not stand out in expression or phosphorylation. Based on these data, we conclude that most HNSCC cell lines express activated EGFR levels driving EGFR dependent cell growth but certain cell lines may have features that weaken the dependence on EGFR. For example, KYSE-70 carries a PIK3CA amplification [21] and KYSE-510 amplifications in c-myc, hst-1 and cyclin D1 [22].

The high specificity of MSD assays for phosphorylation of receptor tyrosine-kinases is exemplified in Supplementary Figure 5. High levels of phosphorylation are observed as a result of RTK amplification and overexpression in few cases which are indication specific like Her2 in breast and gastric cancer, MET in gastric cancer and EGFR in lung and gastric cancer.

### Expression and phosphorylation status of EGFR in clinical HNSCC samples

**Figure 2:**
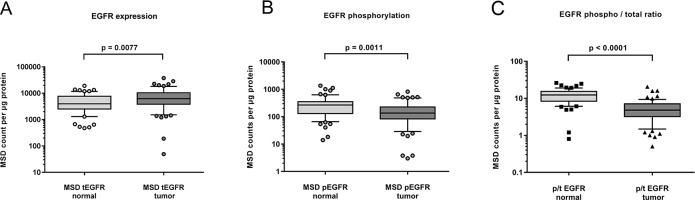
Expression **(A)** and phosphorylation **(B)** of EGFR in fresh frozen samples of HNSCC tumor (n=63) and adjacent normal tissue (n=60). A small but significant increase in EGFR expression was found in tumor samples (A). EGFR phosphorylation however was lower in tumor compared to normal adjacent tissue (B). Shown as phospho- to total ratio, the lack of EGFR activity in HNSCC tumors becomes more evident **(C)**.

We next tested whether the expression and activity level of EGFR in clinical samples of HNSCC is in the range that we observed in the HNSCC cell lines. Samples were obtained during curative surgery for primary HNSCC tumors. Tumors were typically poorly differentiated or undifferentiated, still localized but already with progression to local lymph nodes. The available patient data are summarized in Table 2. For comparison, healthy normal adjacent tissue was also prepared from the surgical safety margin. Figure 2A shows the expression levels of EGFR in tumor and normal tissue. We detected a small but statistically significant overexpression of EGFR in the tumor sample. However, the absolute levels hardly reached the levels observed in HNSCC cell lines. Figure 2B displays the EGFR phosphorylation levels detected in clinical samples of HNSCC. Surprisingly, the activity level was lower in the tumor samples compared to healthy adjacent tissue samples and the absolute counts were low. When expressed as phospho to total ratio, the lower activity level in tumor samples compared to normal adjacent tissue was even more significant (Figure 2C). Neither EGFR expression nor phosphorylation was correlated with tumor differentiation or stage of disease.

**Table 2:** Demographics of the clinical sample donors (the complete clinical data set was available for n=51 of the 63 enrolled patients)

Variable		Number	(%)
Age (median)		66	
Gender	Male	39	76.5
	Female	12	23.5
Tumor Localization	Oral cavity	7	13.7
	Oropharynx	21	41.2
	Hypopharynx	6	11.8
	Larynx	15	29.4
	Other	2	3.9
Tumor Stage	I	6	11.8
	II	1	2.0
	III	6	11.8
	IVa	26	51.0
	IVb	5	9.8
	IVc	7	13.7
Differentiation	Well differentiated	1	2.0
	Moderately differentiated	3	5.9
	Poorly differentiated	35	68.6
	Undifferentiated	12	23.5

### Comparison of EGFR status between cell lines and clinical samples

**Figure 3:**
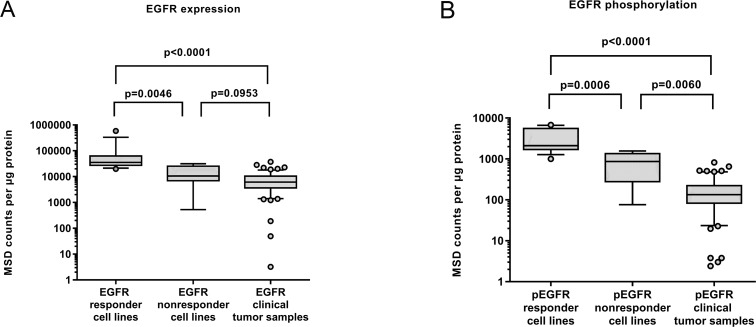
**(A)** Expression and **(B)** phosphorylation of EGFR in cell lines (N=14) classified by their Erlotinib response in comparison to the levels found in HNSCC tumor samples (n=63). It becomes evident that the responding cell lines are not representative for the clinical situation. (Nonresponder cell lines comprise KYSE-70, KYSE-510 and KPL-4 as well as 3 additional MET-dependent cell lines which did not respond to Erlotinib (SNU-1, MKN-45, U-87 MG) in order to gain significant numbers.

In Figure 3 we compare the levels of EGFR expression and phosphorylation between responder and non-responder cell lines and the clinical samples. Non-responders comprise KYSE-70, KYSE-510 and KPL-4 as well as three additional MET-dependent gastric cancer cell lines which did not respond to Erlotinib (SNU-1, MKN-45, U-87MG) in order to gain significant numbers for the analysis. Expression levels in clinical samples were significantly lower than those observed in responder cell lines, they were in the range of non-responding cell lines (Figure 3A). Therefore it seems that most of the established HNSCC cell lines are not representative for the clinical situation. Figure 3B shows that the situation is worse for the tyrosine-1173 phosphorylation: the levels observed in clinical samples were even lower compared to the non-responder cell lines. These findings suggest that these cell lines are not representative for the clinical situation in HNSCC.

### Comparison of EGFR expression in tumor and normal tissue by IHC

As overexpression of EGFR in clinical tumor samples has previously been described, we then investigated EGFR in HNSCC using immunohistochemistry in order to assess the spatial distribution. We first established a staining protocol and controlled the specificity by using formalin-fixed cell pellets of Detroit-562 (highest EGFR expression in MSD), KYSE-510 (lowest EGFR expression) and KPL-4 showing strong, moderate and negative staining (Supplementary Figure 2) proving that the assay is specific and sensitive in the right range of expression levels.

We next stained the formalin fixed HNSCC samples. A broad range of protein expression was detected. The vast majority of clinical samples expressed low to moderate levels of EGFR highly enriched in the plasma membrane. The expression was restricted to the tumor cells and not present in stroma (Supplementary Figure 3). For comparison we also stained the normal adjacent tissue. EGFR was expressed in most of the samples in weak to moderate intensity on the plasma membrane. The expression was restricted to the epithelial layer, most dominant in the basal cell layer and weakening towards the epithelial surface (Supplementary Figure 4).

**Figure 4:**
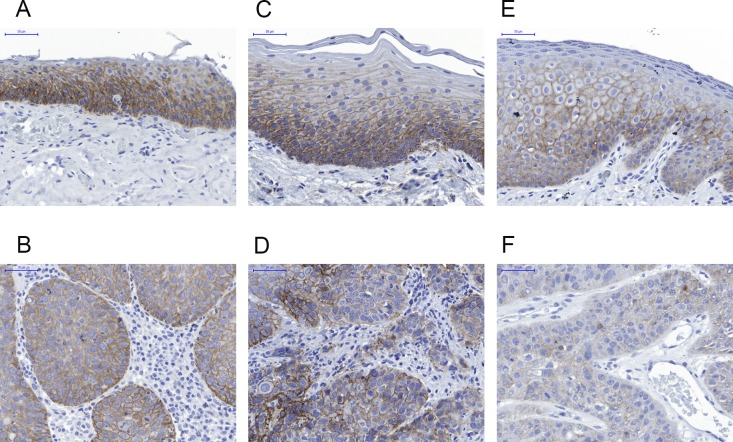
Representative immunohistochemistry results for EGFR expression in normal adjacent tissue (upper row, **A, C, E**) compared to the respective HNSCC lesions in matched samples (lower row **B, D, F**). A and B case 28; C and D case 41; E and F case 63. EGFR is not overexpressed in tumors compared to the basal layer of normal epithelium.

We then compared the EGFR expression in matched pairs of tumor and normal adjacent tissue samples. No trend to increased expression in tumor cells could be detected. Figure 4 shows representative examples of comparisons between normal adjacent (A, C, E) and tumor (B, D, F) tissue of the same donors. Low to moderate expression was detected in the basal to suprabasal layers of the regular epithelium, weakening towards the surface. The underlining connective tissue was negative for EGFR. In the tumor samples weak to moderate signals were detected which were restricted to the tumor cells whereas the surrounding stroma was negative for EGFR expression.

**Figure 5:**
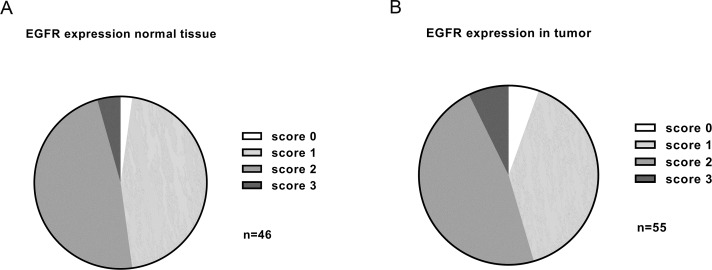
Summary of EGFR expression levels detected in adjacent normal tissue (n=46) **(A)** and HNSCC (n=55) **(B)** by immunohistochemistry. The vast majority of samples had low to moderate EGFR expression. No significant overexpression was found in tumor samples compared to normal tissue (p=0.375).

We then analyzed the complete distribution of staining intensities observed in normal adjacent and tumor samples (Figure 5). Low to moderate expression levels were dominant. No statistically significant difference was found between tumor and normal adjacent tissue samples.

## DISCUSSION

We showed that EGFR is not overexpressed in most clinical HNSCC samples and established cell lines do therefore not represent the general clinical situation. Probably, EGFR-high expressing cells were selected during the establishment of the cell cultures from primary tumors or have evolved in this direction over many years in the cell culture.

Many reviews stress the prognostic value of EGFR expression to support its role as relevant oncogene but only 60% of the studies show association between EGFR expression and outcome whereas 40% do not [1]. Although most reviews claim that EGFR is overexpressed in HNSCC, specific data supporting this are quite limited. The earliest reports often cited are studies that only tested expression on the RNA level. In addition, the RNA expression was not compared to normal adjacent tissue but to a small number of control tissues from patients undergoing surgery for different reasons [23]. Ozanne and Richards described the amplification of EGFR in HNSCC cell lines compared to normal keratinocytes by southern blotting. This was also done for a few patient samples in comparison to normal mucosa showing higher EGFR signals in the HNSCC biopsies [24]. However, no control for adjacent sequences or centromeres was included, so that simple polyploidy cannot be ruled out as a reason. The same group reported increased binding (factor 5) of an iodinated anti-EGFR antibody to cryosections of HNSCC compared to adenocarcinomas and normal mucosa [25]. However, the resolution of this was limited and the normal mucosa showed similarly strong signals with the control antibody raising concerns about the specificity of the approach. Also the original study describing the prognostic value of EGFR levels used the I-125-EGF binding assay in membrane preparations of frozen biopsies without controlling the tumor to stroma ratio [4].

Later studies using immunohistochemistry usually assessed the association of EGFR expression with disease progression. Higher expression by AQUA scores was associated with recurrence and overall survival [26], but no comparison to expression in normal mucosa was described. Sheikh et al. found an association between EGFR expression, nodal status and worse differentiation but time to progression and overall survival did not reach significance [27]. Overexpression was described in this paper but was based on percentage of positive cells not on the intensity of expression. Sheikh et al. also clearly described expression of EGFR in the basal layer of normal epithelium and its extension into the stratum spinosum in hyperplasia. The situation is similar for NSCLC for which the EGFR inhibitors were first developed. The overexpression referred only to the number of positive cells which was higher in tumor as the expression in normal tissue was confined to the basal layer of the bronchioalveolar epithelium. The intense staining of the non-neoplastic epithelium was specifically shown in this paper [28]. EGFR inhibitors were developed based on the prognostic value of EGFR expression and years of clinical studies were required until it was discovered that not the expression of EGFR but activating mutations were predictive for response in NSCLC [29].

We confirm that EGFR expression is maintained in growing keratinocytes and HNSCC cells but lost with differentiation in the normal epithelium. Therefore, the often described overexpression only originated from a larger number of EGFR positive growing cells in tumors and cannot be traced back to an overexpression on the individual cell level. The larger number of EGFR positive cells explains the findings on the level of RNA expression in tumor lysates especially if the stroma content is low in higher grade tumors with worse prognosis.

For the downstream signaling and its effect on cell proliferation and survival the activation of EGFR is much more relevant than the expression level. When we tested the EGFR activation in clinical samples by measuring the phosphorylation of tyrosine-1173 it became clear that in contrast to the expression levels, EGFR phosphorylation was even significantly lower in tumor versus normal adjacent tissue. Strikingly, when compared to established HNSCC cell lines, the EGFR phosphorylation was an order of magnitude lower.

In cell lines, we found EGFR commonly expressed and activated and the majority of cell lines responded to submicromolar concentrations of Erlotinib supporting its potential use in HNSCC. Only two of fourteen HNSCC cell lines did not respond to Erlotinib and these may be driven by other oncogenes as KYSE-70 has an PIK3CA amplification [21] and KYSE-510 bears c-myc, hst-1 and cyclin D1 amplifications [22]. Our finding that most HNSCC cell lines respond to Erlotinib in cell culture confirms earlier findings by Haddad et al., who also showed response in 27 different HNSCC lines. Although there were differences in sensitivity, all cell lines reacted at least in the micromolar concentration range. [16]. The sensitivity was influenced by the epithelial phenotype, resistance caused by EMT as shown by Frederick et al. [15].

Our comparison of cell lines with clinical samples revealed that EGFR expression and activity is more common in cultured cells. A similar finding has been published for phosphotyrosine levels in NSCLC [30] where phosphopeptides from EGFR, MET and EPHA2 were dramatically overrepresented in cell culture samples. Although cell line panels are commonly used to identify predictive biomarkers which would be the famous Achilles heel of cancer [31], it still has to be verified whether the collection of cell lines represents the clinical situation.

The predictive value of cell lines for the clinical situation has been analyzed for several indications [32]. While similarities in expression patterns were reported for breast cancer [33, 34], a greater number of high level amplifications were found in cell lines [35]. A similar finding was reported in ovarian cancer [36]. In HNSCC more mutations were unique to cell lines or tumors than common. Also, the number of amplifications especially in the relevant EGFR, ERBB2 and PIK3CA genes was much higher in cell lines [37].

Therefore, it is important to verify the clinical situation if predictive biomarkers are established based on the properties of cultured cell lines. The prediction of clinical response rates based on response rates obtained in cell line panels is of limited value if the cell lines do not represent the average target expression in the respective human specimen. In addition to the potential differences in responsiveness between cell lines and tumors, the exposures reached in clinical studies may also be the issue. Cell culture conditions differ from the clinical situation, therefore the exposure levels have to be carefully compared. The clinical MTD of EGFR TKIs is limited by skin toxicity reflecting the EGFR expression in the growing keratinocytes. Higher grade skin rash is correlated with response showing that systemic exposure levels are limiting the efficacy at the common dosing regimen. The steady state concentration of 150mg daily Erlotinib was around 2μM [10, 38]. Taking into account that the free fraction of Erlotinib in plasma is only 7%, the active concentration is only reaching approximately 200 nM. Only one cell line in our HNSCC panel would have demonstrated growth inhibition at this concentration. Only the control cell line HCC-827 with an activating EGFR mutation has an IC50 value which would predict a clear response to these drug levels. This situation can be compared to NSCLC. Only a few cell lines were found to be highly sensitive to Gefitinib (with submicromolar IC50s) and the sensitivity correlated with activating mutations and high copy number gains in the EGFR gene. Also, the phosphorylation levels were high in the responder cell lines [39]. The correlation between Erlotinib and Gefitinib sensitivity is excellent as described by Gandhi and colleagues who also showed in a large preclinical study comprising 77 NSCLC lines that all sensitive cell lines (IC50 below 1 μM) had activating mutations or copy number gains but no secondary resistance mutations like T790M or PTEN loss. In that study, six of seven sensitive lines had IC50 values of 200 nM or lower [40]. The biochemical reason for this is the increased affinity for Erlotinib while ATP binding is weakened in the L858R or exon 19 deletions which give the competitive inhibitor a 6 to 130fold advantage relative to the wild-type EGFR [20]. Therefore EGFR mutated cell lines are much more sensitive in proliferation assays [41].

In conclusion, EGFR expression levels alone are not predictive for response to clinically tolerable Erlotinib, concentrations. Only cell lines or tumors with activating mutations seem to be sensitive enough to react to TKI concentrations reached in the clinics.

Therefore, larger studies are required to investigate whether activating mutations or genomic amplifications can be detected in HNSCC patients. The identification of these changes would justify the treatment of biomarker-positive patients with EGFR inhibitors.

## MATERIALS AND METHODS

### Collection of human samples

Sample collection was done in agreement with the declaration of Helsinki 1964. The study was approved by the local ethical committee (EA2/045/10) and respective patient informed consents were obtained before start of the research activities. 63 cases of head and neck cancers of different stages excised during primary surgery were used for this analysis. Samples of tumor and adjacent normal tissue were split and freshly frozen for analysis in sandwich-immunoassays or formalin fixed and paraffin embedded for later use in immunohistochemistry. In some cases no normal adjacent tissue could be obtained or very small samples were lost during processing, therefore numbers of successfully analyzed samples are indicated in the figure legends. Complete clinical data on localization, stage and differentiation of the tumor were available for 51 of the 63 patient samples.

### Tissue culture

All 14 HNSCC and 2 control cell lines were obtained from ATCC or DSMZ and grown in RPMI1640 (Biochrom F1215), Iscove Basal Medium (Biochrom F0465) or DMEM (Gibco 4166-029) supplemented with 10% FCS and 2mM Glutamine according to the instructions of ATCC and DSMZ. Cell were tested and found free of Mycoplasma contamination. Details are described in Supplementary Table 1. No cell line was used above passage 10.

For generation of cell lysates, cells were grown to 70% confluence, washed twice with PBS and directly lysed for 30min on ice using 1mL MSD lysis buffer supplemented with phosphatase inhibitor cocktail (Sigma P0044 and P-5726) and protease inhibitor cocktail (Pierce Biotechnology 87785). Cell lysates were aliquoted, snap frozen in liquid nitrogen and stored at −80°C until used for sandwich-immunoassays.

FFPE pellets were prepared by pelleting the washed cells followed by fixation in 10% neutral buffered formalin for 8 - 18 hours at room temperature. The pellets were stored in 70% ethanol until paraffination using a vacuum infiltrator (Leica ASP200S).

### Immunohistochemistry

Standard procedures were used for immunohistochemistry. In brief, antigen retrieval was performed in pH9 target retrieval solution (Dako S2367) for 17min in a steam cooking device. Endogenous peroxidase activity was blocked with Peroxidase Blocking Solution (Dako S2023) for 15 minutes at room temperature. EGFR receptors were stained using the primary rabbit anti-EGFR antibody clone D38B1 (Cell Signaling Technologies) in 1:900 dilutions in antibody diluent (Dako S2022) for 2h followed by application of the DAKO Envision system (anti-rabbit; Dako K4011) for 1h and addition of the DAB chromophore for 10min. Incubation with antibodies occurred in a humid chamber at room temperature at all times.

### MSD-Immunoassay

EGFR protein expression and phosphorylation of Tyrosine-1173 were analysed by electro-chemiluminescence based sandwich-immunoassays (Mesoscale Discovery, Rockville USA) in lysates made from fresh frozen tumor material or exponentially growing cell cultures according to manufacturer's instructions. Briefly, cells were lysed in MSD lysis buffer with protease inhibitor and phosphatase inhibitors (described above). Protein concentration was determined by BCA (Pierce #23227, Rockford USA). 20 μg of protein content were used per well and results were presented as MSD counts per μg used.

### Proliferation assay

For proliferation assays, cells were seeded at 2000 cells per well in 96 well plates and pre-incubated for 24h in cell culture media. After addition of Erlotinib in different concentrations ranging from 0.15 nM to 3 μM or DMSO control, cells were continuously incubated for 72h before testing viability using the CellTiter-Glo^®^ Luminescent Cell Viability Assay. The maximum concentration of 3 μM was selected because some of the HNSCC cell lines were very sensitive to DMSO and did not tolerate more than 0.1% DMSO final concentration. IC50 and efficacy values were fitted by 4 parameter fit using GraFit Data Analysis Software.

### Statistics

All analyses were performed using GraphPad Prism (La Jolla, CA). For sample sets with Gaussian distribution (IHC scores of clinical samples), the differences between groups were analyzed using student's two-tailed *t*-test paired *t*-test. For sample sets including non-Gaussian distributions (most EGFR expression and phosphorylation data in frozen samples), the Mann-Whitney *U* test was used.

## SUPPLEMENTARY MATERIALS FIGURES AND TABLE




