# Supplementary material for: EGFR overexpression is not common in patients with head and neck cancer. Cell lines are not representative for the clinical situation in this indication
Source: Oncotarget. 2018 Jun 22;9(48):28965–75. doi: 10.18632/oncotarget.25656 (PMC6034751; doi:10.18632/oncotarget.25656)
Supplement: Supplementary file 1 [file oncotarget-09-28965-s001.pdf]

EGFR overexpression is not common in patients with head and neck cancer. Cell lines are not representative for the clinical situation in this indication

SUPPLEMENTARY MATERIALS

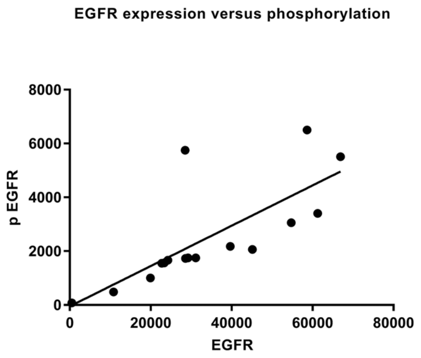

Supplementary Figure 1: Expression and phosphorylation are well correlated (r=0.74) in HNSCC cell lines. The major outlier is the control cell line HCC-827 which is carrying an activating mutation in EGFR.

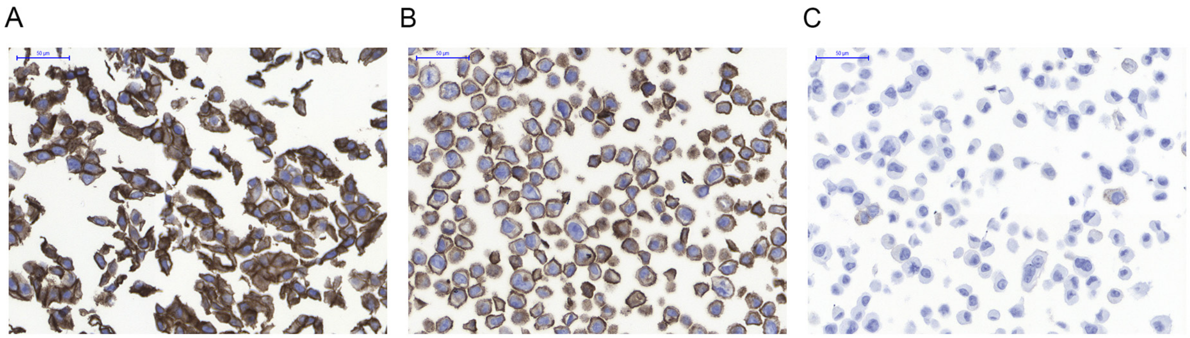

Supplementary Figure 2: Immunohistochemistry results for EGFR expression in control cells. The dynamic range of the established assay is sufficient for the range of EGFR expression observed. Cells of highest (Detroit-562, **A**) and lowest (KYSE-510, **B**) EGFR expression are shown together with the negative control cell line KPL-4 **(C)**.

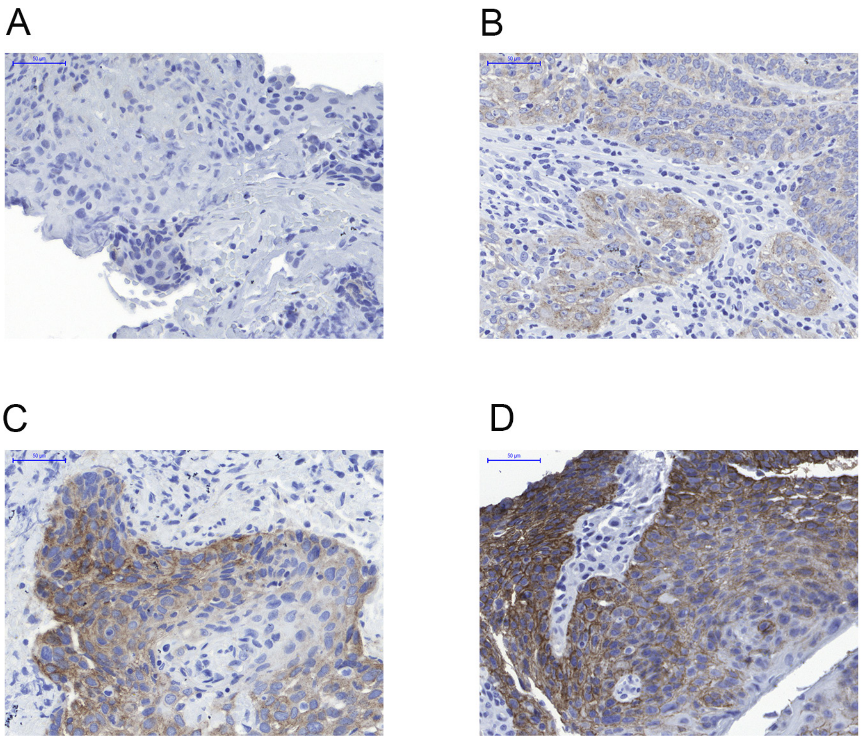

Supplementary Figure 3: Representative immunohistochemistry results for the different levels of EGFR expression in HNSCC lesions. EGFR expression is restricted to well differentiated tumor cells, stroma is free of EGFR expression. **(A)** sample 66T, score 0, **(B)** sample 29T, score1, **(C)** sample 71T, score2 and **(D)** sample 39T, score 3.

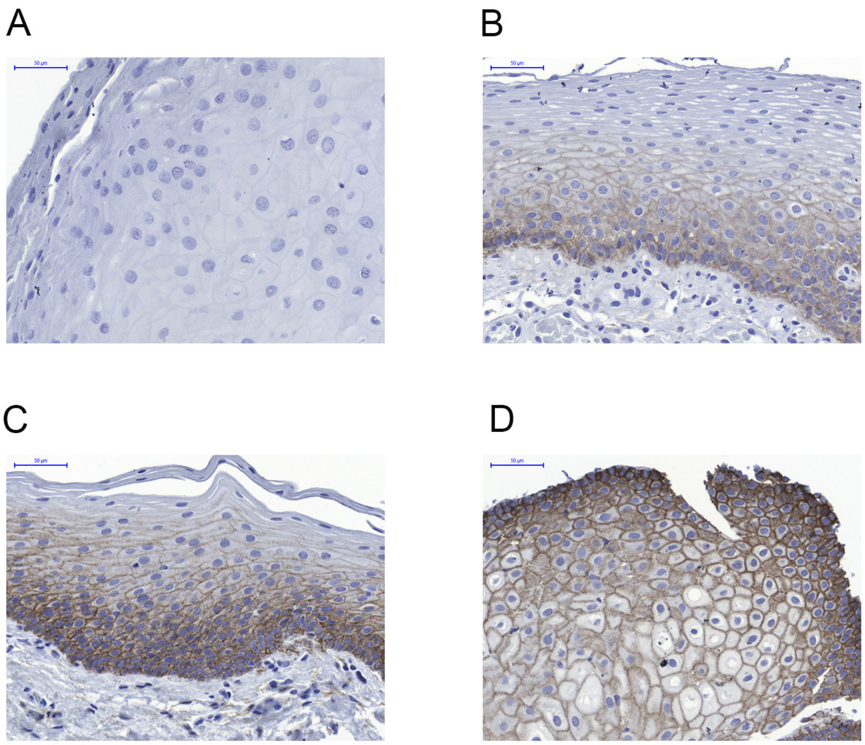

Supplementary Figure 4: Representative immunohistochemistry results for the different levels of EGFR expression in normal adjacent tissue. Moderate to strong staining of the basel and suprabasal layers is the dominant pattern. **(A)** sample 66N, score 0, **(B)** sample 26N, score1, **(C)** sample 41N, score2 and **(D)** sample 35N, score 3.

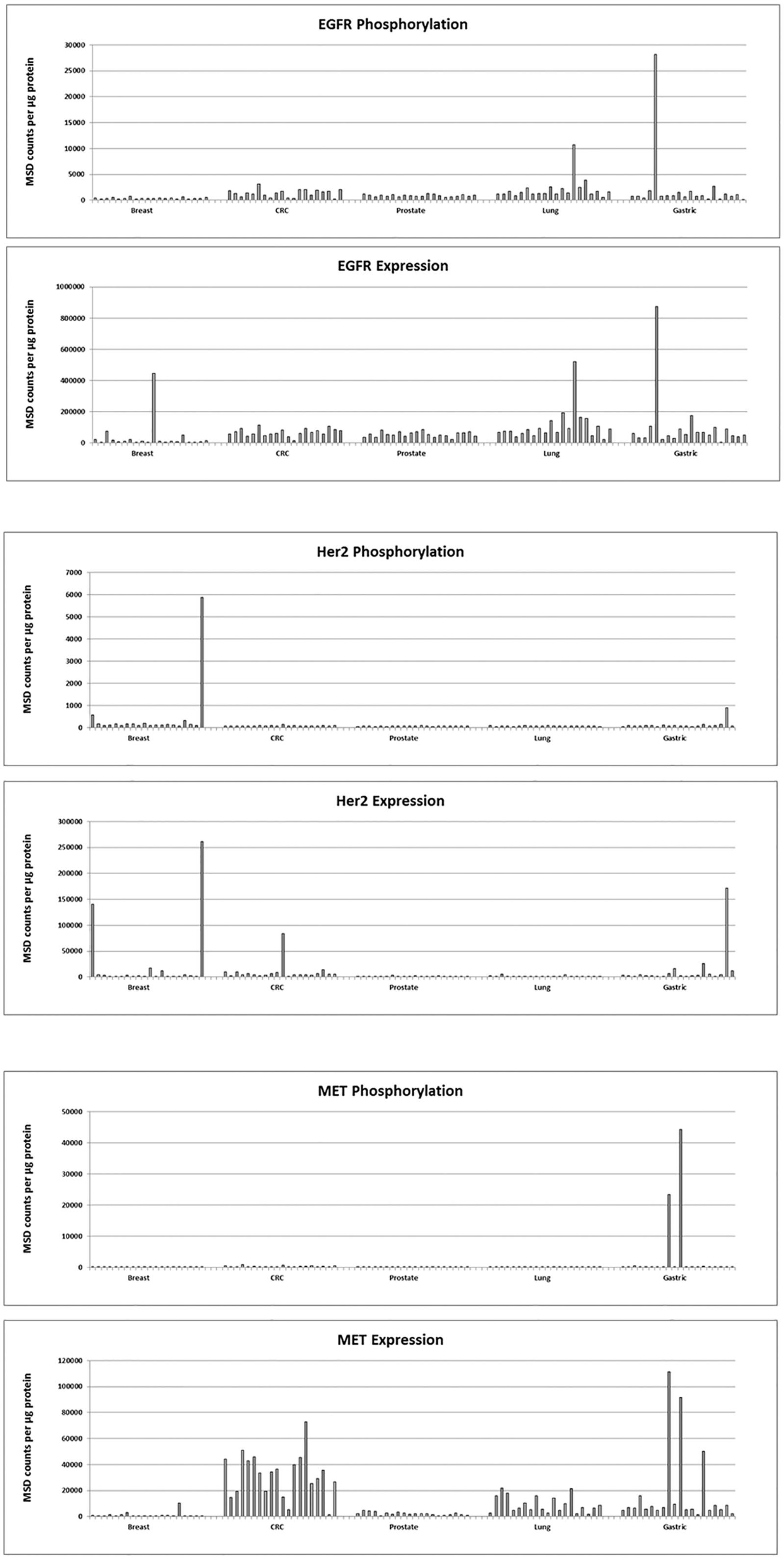

Supplementary Figure 5: 20 fresh frozen samples of each indication were tested for expression and phosphorylation of EGFR, Her2 and MET with the respective MSD assays. The specificity of the phosphorylation is shown by the fact that only cases of high expression display phosphorylation. The data fits well to the known amplification and therapeutic relevance of Her2 in breast and gastric cancer, MET in gastric cancer and EGFR in lung cancer. EGFR amplification has also been described with low prevalence (3.3%).

Supplementary Table 1: Tissue culture conditions and information on the original differentiation status of the used HNSCC cell lines.

See Supplementary File 1
